# ISMB 2003 Text Mining SIG Meeting Report

**DOI:** 10.1002/cfg.338

**Published:** 2003-12

**Authors:** Christian Blaschke, Alexander Yeh, Lynette Hirschman, Alfonso Valencia

**Affiliations:** 1 Protein Design Group CNB/CSIC Madrid Spain; 2 The MITRE Corporation Bedford MA 01730 USA


**Comparative and Functional Genomics**
*Comp Funct Genom* 2003; **4**: 667–673.
Published online in Wiley InterScience (www.interscience.wiley.com). **DOI:** 10.1002/cfg.338

**Conference Review**

### ISMB 2003 Text Mining SIG meeting report

ISMB'03, Brisbane, Australia, 27 June 2003

Christian Blaschke[1]*, Alexander Yeh[2], Lynette Hirschman[2] and Alfonso Valencia[1]

[1] *Protein Design Group, CNB/CSIC, Madrid, Spain*
[2] *The MITRE Corporation, Bedford, MA 01730, USA*

**Correspondence to:*
*Christian Blaschke, Protein Design Group, CNB/CSIC, Madrid, Spain.*
*E-mail: blaschke@cnb.uam.es*

Received: 18 September 2003
Revised: 24 September 2003
Accepted: 25 September 2003

### Introduction

The third meeting of the Special Interest Group (SIG) for Text Mining was held in conjunction with ISMB in Australia this year, following the 2001 meeting in Copenhagen and the 2002 meeting in Edmonton. The Text Mining SIG has been organized by the BioLINK group (**http://www.pdg.cnb.uam.es/BioLINK**), with its main contributors Lynette Hirschman (MITRE, Bedford, MA, USA) and Alfonso Valencia (CNB, Madrid, Spain), together with this year's local organizers Christian Blaschke (CNB), Marc Light (University of Iowa, USA) and Alexander Yeh (MITRE). The SIG's main goal has been to foster communication in text mining and information extraction applied to biology and biomedicine. To this end, the BioLINK group holds regular open meetings to bring together researchers from the field to interchange ideas and share them with a wider community interested in the latest developments. In the past two meetings, the Text Mining SIG has included reports from related SIGs (e.g. BioOntologies and BioPathways).

Information extraction (IE) is an outgrowth of work in automated natural language processing, which began in the 1950s with work on transformational grammar by Zellig Harris and later Noam Chomsky. Information extraction technology made rapid progress starting in the late 1980s, thanks to a series of conferences focused on evaluation of IE: the Message Understanding Conferences (MUCs). There has also been a long history of research on applications in medicine. Applications to the medical field focus on two distinct sub-problems: (a) improved access to the medical literature; and (b) extraction of information from patient records.

Despite the successes in other fields, Natural Language Processing (NLP) techniques were not introduced into biology until the late 1990s. The field has been dominated by two, not necessarily convergent, approaches: (a) application-orientated, where simple methods are used (possibly too simple) to address 'real' biological problems; and (b) tool-orientated, where complex, state-of-the-art NLP methods are used to address problems that are not always relevant to biologists.

During the SIG meeting, it became apparent that three major bottlenecks hinder current development:

1. The complex and non-standardized nomenclature of genes and proteins in the scientific literature. This makes it difficult to identify the basic content of a document, in particular the entities mentioned.

Copyright © 2003 John Wiley & Sons, Ltd.

2. The absence of large, annotated standard corpora for training and evaluation of alternative methods.
3. The lack of common standards and evaluation criteria that allow researchers to compare the performance of different methodologies.

To begin to address these problems, the BioLINK group is organizing a critical assessment of text mining methods later this year (see **http://www.pdg.cnb.uam.es/BioLink/BioCreative.eval.html**). The assessment is inspired by the CASP evaluations and will be carried out in collaboration with SwissProt, HighWire Press, FlyBase and other groups.

### Talks

In this report, we review some of the presentations given at the SIG meeting (Table 1). For more information and copies of the submitted abstracts and presentations, visit **http://www.pdg.cnb.uam.es/BioLink/SpecialInterestTextMining/HANDOUTS/1_BioLINK_handouts_May28.html**.

### Ontologies in Bioinformatics (Robert Stevens)

The basic function of human language is to communicate efficiently (between human beings). To do so, symbols have been created (composed of words) that stand for things. The meaning triangle (Figure 1 [6]) describes the relationship between symbols and things.

Ontologies are conceptual models of the shared and common understanding of a domain and they capture knowledge in a computationally amenable manner. Therefore, they are of specific interest for the NLP community because they provide a

**Table 1.** Talks given at the Text Mining SIG meeting

| Speakers and Affiliation | Title |
|---|---|
| **Robert Stevens.** Univ. Manchester, UK | Report from BioOntologies SIG |
| **Ian Donaldson,** Joel Martin, Berry de Bruijn, Christopher W.V. Hogue. Univ. Toronto, Canada | PreBIND and Textomy—mining the biomedical literature for protein–protein interactions using a support vector machine |
| **Andy Fulmer,** Jun Xu, Steven Zhao. Procter & Gamble, USA | An overview of text mining in the biology domain at P&G |
| George Demetriou, **Robert Gaizauskas**. Univ. Sheffield, UK | Corpus resources for development and evaluation of a biological text mining system |
| Yuka Tateisi, Tomoko Ohta, **Jin-dong Kim**, Huaquing Hong, Su Jian, Jun-ichi Tsujii. CREST, Japan Science and Technology Corporation | The GENIA corpus: MEDLINE abstracts annotated with linguistic information |
| Seth Kulick, Mark Liberman, **Andrew Schein.** Univ. Pennsylvania, USA | Shallow semantic annotation of biomedical corpora for information extraction |
| **Maria Samsonova.** St.Petersburg State Polytechnical University, Russia | Processing of the natural language queries to a relational database |
| Tony C. Smith, **John G. Cleary.** Univ. Waikato, New Zealand | Automatically linking MEDLINE abstracts to the Gene Ontology |
| Yoshimasa Tsuruoka, **Teruyoshi Hishiki**, Osamu Ogasawara, Kousaku Okubo. CREST, Japan Science and Technology Corporation | Integration of diverse knowledge and data into biomedical knowledge matrices |
| Eunji Yi, **Gary G. Lee,** Soo-Jun Park. Pohang Univ. Science and Technology, Korea | HMM-based protein name recognition with edit distance using automatically annotated corpus |
| **Francisco M. Couto,** Mario J. Silva, Pedro Coutinho. Univ Lisbon, Portugal | Curating extracted information through the correlation between structure and function |
| **Rune Linding,** Peter O'Hanlon, **Ulrich Reincke,** Toby Gibson. EMBL, Heidelberg, Germany | Profiling and classification of scientific documents with SAS Text Miner |
| **Alex Yeh,** Lynette Hirschman, Alex Morgan. MITRE, USA | BioCreAtIvE: entity extraction |
| **Christian Blaschke,** Alfonso Valencia. CNB, Spain | BioCreAtIvE: functional extraction |

Bold type indicates the person who spoke at the meeting.

Copyright © 2003 John Wiley & Sons, Ltd. 

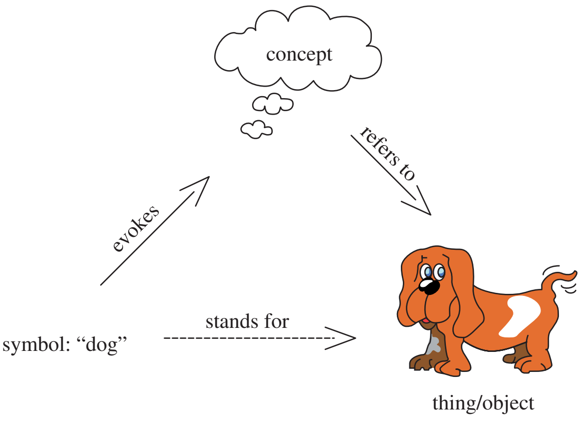

**Figure 1.** The meaning triangle describes how symbols (part of the human language), concepts (abstract meanings) and things (real world objects) are related framework into which information extracted from text can be mapped. On the other hand, text analysis can support building ontologies by making information in the literature more easily accessible.

There are a number of ontologies that are of specific interest to biology, e.g. the Gene Ontology (GO [1]), the Disease Ontology (DO; **http://diseaseontology.sourceforge.net/**), the Mouse Anatomy Ontology (**http://www.informatics.jax.org/searches/anatdict_form.shtml**), the ontology of *E. coli* metabolism (EcoCyc [4]), TAMBIS [7] and PharmGKB (**http://www.pharmgkb.org/**).

The GO ontology covers molecular function, biological process and cellular components for gene products. The first release consisted of about 3500 terms; it now contains around 15 000 terms and is still growing. Currently it covers some 15 (model) organisms. GO has been criticized (mainly by non-biologists) because of its *ad hoc* construction and impoverished model of relationships: it contains only 'is-a' and 'part-of ' relationships. However, its success and wide use has proved that the important issues for an ontology are that it contain useful knowledge, modelled in a way that can be easily applied.

### Protein name detection (Eunji Yi)

Before knowledge models such as ontologies can be applied to text analysis, one first has to detect the basic symbols (named entities) that represent the concepts in the text. In the biomedical domain, protein names are among the concepts of central importance but they are notoriously difficult to detect because of the absence of an accepted (and used) standard nomenclature. There have been two approaches for the detection of protein names in scientific text: machine learning methods and rule-based systems.

Machine learning approaches suffer from the lack of (large) annotated corpora for training and the excessive spelling variations in names. Yi *et al.* used a hidden Markov model (HMM)-based system and showed that training corpora can be created automatically from MedLine (**http://www.ncbi.nlm.nih.gov/entrez/query.fcgi?db = PubMed**) abstracts tagged with protein names extracted from Swiss-Prot [2]. Their results indicate that systems trained on such corpora do not suffer in precision and show improvements in recall. This makes automatically annotated corpora an interesting solution when sufficiently large hand-created datasets are not available.

The authors propose the use of a distance measure between strings (edit distance, introduced by Wagner and Fisher [8]) integrated in the HMM to allow shallow matching between strings to account for spelling variants. As expected, this extension increases the recall of the system but precision suffers somewhat.

In conclusion, automatically annotated training corpora and relaxation in string matching improve the performance of machine learning methods in the task of protein name detection in scientific text.

### Text mining at P & G (Andrew Fulmer)

Andrew Fulmer from Procter & Gamble gave insights in how text mining is used in the context of a pharmaceutical company. P&G conducts research in biology to support its businesses in pharmaceutical drugs, personal health care, pet care and consumer products. They have established the capabilities to conduct high-throughput Affymetrix gene chip expression studies, where a single experiment generates data on ~10 000 different genes. The demand to interpret these datasets in a timely manner has motivated their entry into the text mining field.

In 1999, in collaboration with scientists at Iowa State University, P&G started to develop PathBinder, which is now used to harvest signal

Copyright © 2003 John Wiley & Sons, Ltd.

transduction–gene regulatory pathway interactions. These interactions are curated by their project biologists into a pathways knowledge base, built on a simple logical interaction model, with a separate suite of tools to build and analyse pathways. To increase recall, better NLP filters to post-process the PathBinder sentences are now being developed with a group of linguists at Los Alamos National Labs.

In 2001, with the initial pathway text mining initiatives under way, more attention was paid to mining 'functional context' to characterize members of a statistically filtered list of genes from a gene chip study. A gene chip experiment involves about 10 000 genes, which are filtered down to ∼1000 genes by statistical data analysis and external information stemming from the experimental design. To discover a 'useful story' and create a biological model, this number has to be reduced by another one to two orders of magnitude. One approach is to develop tools to annotate the genes with GO terms, using information mined from Medline, then 'cluster' the gene list in ontology space. An ontology clustering tool is under development with the Los Alamos National Labs, while the GOMedlineMiner is still 'in the intellectual incubator'.

### PreBIND and Textomy (Ian Donaldson)

The majority of experimentally verified molecular interaction and biological pathway data are present in the unstructured text of biomedical journal articles where they are inaccessible to computational methods. The Biomolecular Interaction Network Database (BIND) is a curated catalogue of biomolecular interactions, complexes and pathways and seeks to capture these data in a machine-readable format. It currently contains about 17 000 protein–protein interactions, ∼48 000 protein–small molecule interactions, ∼1300 molecular complexes and eight pathways.

PreBIND and Textomy are two components of a literature-mining system designed to find protein–protein interaction information and present this to curators or public users for review and submission to the BIND database. This system couples a co-occurrence network of protein names with Support Vector Machine (SVM) technology that identifies abstracts describing biomolecular interactions.

Performance analyses estimated that the SVM F-measure was 92% and that the system would be able to recall up to 60% of all non-high-throughput interactions present in the MIPS yeast–protein interaction database. Finally, this system was applied to a real-world curation problem and its use was found to reduce the task duration by 70%.

Machine learning methods are useful as tools to direct interaction and pathway database backfilling; however, this potential can only be realized if these techniques are coupled with human review and entry into a factual database such as BIND. The PreBIND system described here is available to the public. Current capabilities allow searching for human, mouse and yeast protein-interaction information.

### Corpus work (Andrew Schein, Robert Gaizauskas, Jin-dong Kim)

It is generally accepted that the free availability of suitably annotated text corpora is a prerequisite for development and evaluation of language processing systems. Such corpora are linguistic resources fundamental for a number of tasks:

1. *Definition of a text analysis task*. Annotating text for a specific purpose aids in defining the task more precisely, it shows which entities must be taken into account, which relationships exist between them, and it encourages refinement of the task guidelines.
2. *System development*. Two basic methods exist for building NLP systems: hand-crafted rule-based methods or machine learning (ML)-based methods that make heavy use of statistics and pattern analysis. ML methods depend on suitable training corpora (in general the larger the better) for setting up the system.
3. *System evaluation*. To assess performance, different NLP systems are applied to the same corpora and the results are compared. The lack of freely available corpora that are sufficiently large and general in focus has hindered the comparison of NLP systems applied to biology.

The different works presented at the SIG meeting highlighted a number of technical and theoretical issues that have to be taken into account for building annotated text corpora:

Copyright © 2003 John Wiley & Sons, Ltd. 

1. Definition of the 'correct' annotation scheme. The scheme has to be specific enough to be useful, but not overly specific (trying to capture more information than is actually in the text).
2. The previous point influences the level of inter-annotator agreement. The more specific the scheme, the less the annotators will agree on how to annotate a specific text and no homogeneous results will be produced.
3. The tension between the domain experts and computational linguists needs to be resolved. Domain experts want to capture some kind of information, but have limited understanding of the linguistic basics (they tend to choose computationally impractical solutions); while the computational linguists have limited domain knowledge and tend to create systems that are theoretically elegant but difficult to use by the domain experts.
4. Text corpora can be annotated at very different levels:
   i. Part-of-speech (POS) of words.
   ii. Entities (e.g. genes, proteins, chemical substances, diseases, protein structure elements, etc.).
   iii. Partial or full parse-tree structures to express relations between the tagged entities or more general subject–object relationships.
   iv. Co-references (pronouns like 'it' or 'they' that refer to something explicitly expressed earlier in the text).
5. Evaluation tools developed for different domains have to be adapted to the biological literature.

Kulick *et al.* are developing new linguistic resources in three categories: a large corpus of biomedical text annotated with syntactic structure (Treebank [5]) and predicate–argument structure ('proposition bank' or Propbank); a large set of biomedical abstracts and full-text articles annotated with entities and relations of interest to researchers, such as enzyme inhibition, or mutation/cancer connections (Factbanks); and broad-coverage lexicons and tools for the analysis of biomedical texts. Furthermore, they are developing and adapting software tools that allow human experts to annotate biomedical texts for entity tagging, as well as for treebanking and propbanking. The project focuses initially on two applications: drug development (in collaboration with researchers in the Knowledge Integration and Discovery Systems group at GlaxoSmithKline) and paediatric oncology (in collaboration with researchers in the eGenome group at Children's Hospital of Philadelphia). These applications, worthwhile in their own right, provide excellent test beds for broader research efforts in natural language processing and data integration.

Tateisi *et al.* are developing the GENIA corpus as part of a project for building NLP systems for information extraction of biological reactions. The corpus is a collection of articles concerning the reactions of transcription factors in human blood cells, extracted from MEDLINE, which are annotated in XML format. The first focus was to annotate the actors of biological events in terms of an ontology specifically created for the project; the ontology includes organic and inorganic substances, nucleic acids and proteins and also classes for the place of action such as cell component, tissue or body parts. The authors also reported on current developments to annotate part-of-speech, (partial) parse tree structures and co-references.

Demetriou and Gaizauskas reported on the development of the PASTA system. PASTA, for Protein Active Site Template Acquisition system, is a text mining system for the automatic extraction of information relating to protein structures from the biological literature. A corpus of texts relevant to the study of protein structures was assembled. The primary source of information for retrieving Medline abstracts relevant to protein structures was the Protein Data Bank (PDB [3]). The papers for all macromolecular structures deposited in the PDB during the years 1994–1998 were extracted from Medline. The final corpus consisted of 1514 abstracts, with 414 257 words. Texts were annotated with terminology class information (e.g. species, regions in protein structures, secondary structures, residues, etc.) in SGML markup and a number of templates to capture key information about protein structure, in the style of MUC (**http://www-nlpir.nist.gov/related_projects/muc/proceedings/muc_7_toc.html**), were defined. The resources that were created during the PASTA project are freely available.

Copyright © 2003 John Wiley & Sons, Ltd. 

### Commercial systems (John G. Cleary, Rune Linding, Ulrich Reincke)

In recent years, companies have been developing NLP systems for biology. These efforts are now bearing fruit and the first commercial systems (although rather narrow in their focus) are entering the market.

Smith and Cleary presented the 'Gene Ontology Knowledge Discovery System' (GO-KDS), a publicly available web application that automatically connects biomedical documents to terms from the Gene Ontology, thereby amplifying the potential of GO to elucidate the knowledge embedded within biomedical literature. GO-KDS uses machine learning techniques to infer general semantic models for each GO term from training documents gleaned from the references available in public gene/protein databases. The expert gives the learning algorithm some number of documents deemed exemplars of a particular semantic class. The algorithm identifies all salient features of the documents and weights those that are the best indicators for determining whether or not each document is an instance of the concept being learned. The result is a characteristic computer model that can subsequently be used to predict how likely it is that any future novel document also belongs in that semantic class and to classify these abstracts to appropriate GO terms.

Linding *et al.* presented a cooperative work between the SAS Institute and the ELM Consortium at the European Molecular Biology Laboratory on the development of a text mining-application for the automated identification and ranking of scientific articles. The 'topic scoring' engine is based on the SAS Text Miner™. The topic scoring engine identifies documents with similar content and creates search-profiles to capture the congruencies of the documents. The topic scoring engine replaces keyword querying of bibliographic databases, such as PubMed, with a structured automated process by means of a 'document-based retrieval'. This will reduce research time while improving the quality of the results. The topic scoring engine does not look for a pre-defined vocabulary (which is what a search engine would do) but tests, with different types of singular value decompositions, all possible information resolutions of the concepts underlying the text. These profiles are subsequently applied as filters to new publications. This allows the user to seek publications matching these profiles without having to submit complex queries. SAS and EMBL plan to provide this as a public service to the scientific community after a trial period.

### BioCreative: evaluation of text mining systems (Alexander Yeh, Christian Blaschke)

Many groups are now working in the area of text mining. However, despite the increased activity in this area, the absence of common standards and evaluation criteria has resulted in a situation in which it is not possible to compare the different approaches because the various groups involved are addressing different problems, often using private data sets. As a result, it is impossible to determine how good the existing systems are and what performance can be expected in real applications.

This is similar to the situation in text processing in the early 1990s, prior to the introduction of the Message Understanding Conferences (MUC). With the introduction of a common evaluation and standardized evaluation metrics, it became possible to compare approaches, to assess which techniques did and did not work, and to make progress. This progress resulted in the creation of standard tools available to the general research community. The field of biology is ripe for a similar experiment.

Therefore, the BioLINK group (Biological Literature, Information and Knowledge, **http://www.pdg.cnb.uam.es/BioLINK/**) is organizing a CASP-like evaluation for the text data mining community applied to biology. The two main tasks specifically address the currently existing bottlenecks in the field: (a) the correct detection of gene and protein names in text (named 'entity detection'); and (b) the extraction of functional information related to proteins based on the GO classification system, based on full-text documents.

### Acknowledgements

The organizers of the workshop would like to thank especially all the speakers for their presentations, Marc Light for co-organizing the event, Steven Leard and the ISCB (The International Society for Computational Biology) for organizing the logistics and the infrastructure, and the EU projects ORIEL (Contract number: IST-2001-32688) and TEMBLOR (Contract number: VEQLRT 2001 00015) for financial support.

Copyright © 2003 John Wiley & Sons, Ltd. 

### References

1. Ashburner M, Ball CA, Blake JA *et al*. 2000. Gene ontology: tool for the unification of biology. *Nature Genet* **25**: 25–29.
2. Bairoch A, Apweiler R. 2000. The SWISS-PROT protein sequence database and its supplement TrEMBL. *Nucleic Acids Res* **28**: 45–48.
3. Berman HM, Westbrook J, Feng Z *et al*. 2000. The protein data bank. *Nucleic Acids Res* **28**: 235–242.
4. Karp PD, Riley M, Saier M, *et al*. 2002. The Ecocyc Database. *Nucleic Acids Res* **30**: 56–58.
5. Marcus MP, Santorini B, Marcinkiewicz MA. 1993. Building a large annotated corpus of English: the Penn treebank. *Comput Linguist* **19**: 313–330.
6. Ogden C, Richards I. 1923. *The Meaning of Meaning: A Study of the Influence of Language upon Thought and of the Science of Symbolism*. Routledge & Kegan Paul: London.
7. Stevens R, Baker P, Bechhofer S, *et al*. 2000. TAMBIS: transparent access to multiple bioinformatics information sources. *Bioinformatics* **16**: 184–186.
8. Wagner RA, Fisher MJ. 1974. The string to string correction problem. *J Assoc Comput Mach* **21**: 168–173.

Copyright © 2003 John Wiley & Sons, Ltd.
